# A context-specific evaluation of polygenic embryo screening in best-prognosis preimplantation genetic testing cycles

**DOI:** 10.1093/hropen/hoag078

**Published:** 2026-09-08

**Authors:** Qi Wang, SiCong Zeng, ZiXu Chen, Liang Hu, Xiao Hu, PingYuan Xie, Juan Du, XiLin Xu, GuangXiu Lu, YueQiu Tan, Ge Lin

**Affiliations:** Clinical Research Center for Reproduction and Genetics in Hunan Province, Reproductive & Genetic Hospital of CITIC-Xiangya, Changsha, China; Clinical Research Center for Reproduction and Genetics in Hunan Province, Reproductive & Genetic Hospital of CITIC-Xiangya, Changsha, China; Clinical Research Center for Reproduction and Genetics in Hunan Province, Reproductive & Genetic Hospital of CITIC-Xiangya, Changsha, China; NHC Key Laboratory of Human Stem Cell and Reproductive Engineering, Xiangya School of Basic Medical Sciences, Central South University, Changsha, China; Clinical Research Center for Reproduction and Genetics in Hunan Province, Reproductive & Genetic Hospital of CITIC-Xiangya, Changsha, China; NHC Key Laboratory of Human Stem Cell and Reproductive Engineering, Xiangya School of Basic Medical Sciences, Central South University, Changsha, China; Clinical Research Center for Reproduction and Genetics in Hunan Province, Reproductive & Genetic Hospital of CITIC-Xiangya, Changsha, China; Clinical Research Center for Reproduction and Genetics in Hunan Province, Reproductive & Genetic Hospital of CITIC-Xiangya, Changsha, China; Clinical Research Center for Reproduction and Genetics in Hunan Province, Reproductive & Genetic Hospital of CITIC-Xiangya, Changsha, China; NHC Key Laboratory of Human Stem Cell and Reproductive Engineering, Xiangya School of Basic Medical Sciences, Central South University, Changsha, China; Clinical Research Center for Reproduction and Genetics in Hunan Province, Reproductive & Genetic Hospital of CITIC-Xiangya, Changsha, China; Clinical Research Center for Reproduction and Genetics in Hunan Province, Reproductive & Genetic Hospital of CITIC-Xiangya, Changsha, China; Clinical Research Center for Reproduction and Genetics in Hunan Province, Reproductive & Genetic Hospital of CITIC-Xiangya, Changsha, China; NHC Key Laboratory of Human Stem Cell and Reproductive Engineering, Xiangya School of Basic Medical Sciences, Central South University, Changsha, China; Clinical Research Center for Reproduction and Genetics in Hunan Province, Reproductive & Genetic Hospital of CITIC-Xiangya, Changsha, China; NHC Key Laboratory of Human Stem Cell and Reproductive Engineering, Xiangya School of Basic Medical Sciences, Central South University, Changsha, China

**Keywords:** polygenic embryo screening, PES, preimplantation genetic testing for monogenic disorders (PGT-M), breast cancer, polygenic information, IVF cycle

## Abstract

**STUDY QUESTION:**

In best-prognosis preimplantation genetic testing (PGT) cycles—defined by the availability of multiple transferable embryos—can polygenic embryo screening (PES) provide meaningful, context-dependent stratification that may influence embryo prioritization?

**SUMMARY ANSWER:**

In a sister-pair breast cancer validation cohort, the polygenic risk score (PRS) showed modest predictive performance, and when applied retrospectively to embryos, differences in modelled susceptibility were context-dependent, primarily influenced by the number of available embryos, parental polygenic risk profile, the limited discriminative power of the PRS, and the presence of disease-related monogenic variants.

**WHAT IS KNOWN ALREADY:**

The use of PES as an embryo-ranking tool within PGT remains controversial. Its potential utility depends on embryo availability and clinical context, yet empirical evaluations in real-world PGT settings remain limited.

**STUDY DESIGN, SIZE, DURATION:**

This retrospective study combined (i) family-based validation using 184 affected–unaffected sisters, and (ii) embryo-level analysis of 310 PGT for monogenic disorders (PGT-M) cycles comprising 1722 embryos. Simulation modelling estimated differences in modelled embryo-level PRS percentile ranking under varying clinical and genetic scenarios.

**PARTICIPANTS/MATERIALS, SETTING, METHODS:**

Family-based performance of a breast cancer PRS was evaluated using affected and unaffected sister pairs. Embryo genotype data from PGT-M cycles were used to model PES under varying conditions, including the number of transferable embryos, the presence of pathogenic monogenic variants, and parental PRS percentile strata.

**MAIN RESULTS AND THE ROLE OF CHANCE:**

Within-family PRS discrimination was modest: sisters in the top 10% of the PRS distribution had 4.07-fold higher breast cancer odds than their siblings (95% CI: 1.55–6.33). In the PGT-M cohort, 55% of cycles produced ≥3 transferable embryos, with a median within-cycle spread of approximately 25 PRS percentiles. In *BRCA1/2*-related cycles, PRS did not introduce distinct risk strata but revealed dispersion in modelled susceptibility among embryos sharing the same monogenic background. Embryo PRS distributions were concordant with parental profiles: 71% of embryos from couples with parental PRS ≥80th percentile were classified as high-PRS, compared with 7% when at least one parent was ≤20th percentile. Simulation analyses indicated that PRS-based embryo prioritization was associated with a 12-point lower mean embryo PRS percentile compared with morphology-based embryo prioritization in PGT-M cycles, attenuating to 10 points when PGT-A was incorporated. These differences reflect a statistical surrogate outcome only and do not demonstrate reduction in lifetime disease incidence or other clinical benefits.

**LARGE SCALE DATA:**

N/A

**LIMITATIONS, REASONS FOR CAUTION:**

These findings derive from high-prognosis PGT-M cycles and may not generalize to other PGT settings. Current PRS models have modest performance and variable transferability across ancestries. The retrospective design, limited subgroup sizes, and modelling assumptions (e.g. aneuploidy and sex distribution) further constrain interpretation. The primary simulation outcome—PRS percentile shift—is a hypothesis-generating surrogate endpoint, not a clinical outcome; clinical benefit was not assessed or demonstrated. These findings should not be interpreted as supporting routine clinical implementation of PES; premature use may increase parental anxiety, inequitable access, and pressure to rank embryos using predictions that remain unvalidated at the embryo level.

**WIDER IMPLICATIONS OF THE FINDINGS:**

This study provides an empirical framework for evaluating the current limits of PES within PGT. The observed PRS percentile shifts appear concentrated in best-prognosis cycles with multiple transferable embryos and elevated parental PRS, whereas little change is observed when embryo numbers are limited or risk is dominated by high-penetrance monogenic variants. These findings are hypothesis-generating and may inform future prospective research and ethical discussion, while underscoring that clinical utility and implementation require further validation within appropriately governed settings.

**FUNDING:**

This study was funded by Major Scientific Program of CITIC Group (No. 2023ZXKYB34100), the Science Foundation of Hunan Province (Grant 2023JJ30422), and Health Research Project of Hunan Provincial Health Commission (grant number: W20243089).

**DISCLOSURES:**

The authors declare no conflicts of interest.

WHAT DOES THIS MEAN FOR PATIENTS? Polygenic embryo screening combines information from many genetic differences that each have a small effect to estimate whether an embryo may have a higher or lower inherited chance of some common diseases later in life. On its own, it is not recommended as a routine way to choose embryos, because long-term effects on a child’s health have not yet been established, the scores work less well among family members than in the general population, have not been fully tested at the embryo level, and are strongly influenced by the parents’ own genetic background.

In this study of breast cancer risk scores in families and in embryos from couples having preimplantation genetic testing (PGT) for serious inherited conditions, differences between embryos were usually modest, depended on how many embryos were available, and mattered much less when risk was mainly due to a strong inherited mutation. Using a research model—not real clinical treatment—we compared choosing embryos by genetic risk score versus standard embryo quality grading. The embryo selected under the two approaches typically differed by only about 10–12 points in score ranking; whether this would meaningfully change a child’s future health remains unknown.

These findings do not support using polygenic screening on its own in everyday clinical care. Where it is considered at all, any role would be limited and only as a possible add-on within established PGT, after harmful gene changes and chromosome problems are excluded and when several embryos are available. This remains a research-stage approach and should not replace standard PGT. Some companies already offer similar testing commercially; our results should not be taken to justify standalone or routine use outside properly validated research settings.

## Introduction

Preimplantation genetic testing (PGT) enables the detection of genetic variants in embryos prior to implantation and is intrinsically linked to IVF procedures (ASRM Ethics and Practice Committees, 2025). PGT includes three subcategories: PGT-A (aneuploidy screening), PGT-M (monogenic disorder testing), and PGT-SR (structural rearrangement testing). Within this framework, embryos are selected for transfer based on their genetic profiles, thereby avoiding the implantation of embryos with known pathogenic genotypes (Penzias *et al.*, 2018; Harper *et al.*, 2021), which provides a robust and clinically established preventive strategy.

Advances in genome-wide association studies (GWAS) have enabled the development of polygenic risk scores (PRS), which estimate susceptibility by aggregating the small effects of numerous common variants. Although predictive performance—typically assessed by the area under the receiver operating curve (AUROC)—is modest (generally 0.6–0.7), PRS can still support meaningful risk stratification. Their additive structure produces a continuous risk distribution across the population, resulting in several-fold differences in relative risk between individuals at opposite extremes (e.g. top vs bottom 20th percentile). Thus, PRS represents a probabilistic tool for population-level stratification rather than deterministic predictors of individual outcomes. This raises an important question: could such probabilistic information be extended to guide embryo selection during IVF?

Polygenic embryo screening (PES) has been proposed as an extension of PRS to the embryo level, enabling relative risk ranking among embryos from the same couple. However, PES remains scientifically and ethically debated (Treff *et al.*, 2020; Siermann *et al.*, 2022b; Capalbo *et al.*, 2024). Integrating PES into PGT workflows may offer a more ethically constrained model, in which polygenic information is used only to rank embryos after exclusion of certain abnormalities, rather than to discard embryos solely on the basis of polygenic risk (Treff *et al.*, 2019a; Capalbo *et al.*, 2024). Such a ‘ranking-only’ strategy may preserve parental autonomy while maximizing the use of transferable embryos within a PGT cycle.

Importantly, PES is only meaningful when multiple embryos are available for comparison. Compared with PGT-A (often indicated for advanced maternal age) and PGT-SR (often indicated for structural chromosomal rearrangements), PGT-M cycles typically yield a higher proportion of embryos free of pathogenic variants and suitable for transfer (ESHRE PGT-M Working Group *et al.*, 2020). In addition, PGT-M routinely incorporates both direct mutation testing and linkage analysis, generating richer molecular data than PGT-A or PGT-SR, which focus primarily on chromosomal assessment. Integrating PES into PGT-M workflows therefore largely leverages existing genetic information and imposes minimal additional testing burden. Under these conditions, high-prognosis PGT-M cycles may provide a conceptually appropriate context for evaluating PES within a research framework.

Despite these conceptual advantages, the scientific validity and clinical significance of PES remain uncertain. Current PRS models show limited discrimination within families, exhibit population-specific biases, and lack robust validation at the sibling or embryo level. Concerns also persist regarding potential misuse for non-disease trait selection (Karavani *et al.*, 2019; Treff *et al.*, 2019b). Moreover, most prior evaluations rely on simulation studies assuming large or idealized embryo cohorts. Such assumptions differ from routine PGT practice, where embryo numbers are limited, parental polygenic backgrounds vary, and monogenic variants may coexist. Under these clinically realistic conditions, the significance of PRS-based embryo ranking within PGT-M workflows remains unclear.

To address these gaps, we focused on breast cancer, whose PRS models are among the most extensively validated and are currently undergoing evaluation in adult populations, with encouraging early results (Mavaddat *et al.*, 2019; Kramer *et al.*, 2020; Ho *et al.*, 2022). Restricting analysis to a single high-burden disease aligns with professional guidance (ASRM Ethics and Practice Committees, 2025) recommending that early PES evaluations be limited to serious conditions supported by robust genetic evidence and plausible clinical utility, rather than non-disease traits. Breast cancer therefore provides an ethically defensible and scientifically informative model.

We validated an East Asian-specific 263 single-nucleotide polymorphisms (SNPs) breast cancer PRS in a sister-pair cohort. We then retrospectively applied the model to PGT-M families, and simulated embryo selection scenarios to quantify differences in PRS-based embryo ranking. By estimating the magnitude and constraints of these ranking differences, this study provides empirical data to inform the evaluation of PES within PGT-M workflows and to clarify the practical and ethical boundaries of further research.

## Materials and methods

### Ethical approval

This study was approved by the institutional ethics committee of the Reproductive Genetic Hospital of CITIC Xiangya (Approval No. LL-SC-2023-040).

### Study participants

The two primary datasets used in this study were a sister cohort of local Han Chinese individuals, which was employed to evaluate model performance, and a PGT-M cohort. Participants provided written informed consent, all studies were approved by the relevant ethics committee, and all procedures were conducted in accordance with the ethical standards of these committees.

In the sister cohort dataset, we enrolled 184 local Han Chinese sisters to validate PRS performance (74 breast cancer cases and 110 unaffected sisters). Eligible pairs comprised one sister with histologically confirmed breast cancer and one cancer‑free sister, and the unaffected sister was older than the case at time of diagnosis. Cases were diagnosed at a mean age of 54.1 ± 6.9 years; whereas unaffected sisters had a mean age 65.8 ± 4.4 years. All participants were evaluated and tested negative for known breast cancer susceptibility gene mutations. Participant characteristics included mean age of 59.0 ± 6.0 years, mean age at menarche 14.7 ± 2.0 years, mean BMI 23.9 ± 3.2 kg/m², and 97.3% postmenopausal.

The full PGT-M cohort comprised 416 cycles involving 2010 embryos from cycles treated at CITIC-Xiangya Reproductive and Genetics Hospital between January 2021 and December 2024. After excluding cycles with incomplete parental information, 310 PGT-M cycles comprising 1722 embryos were retained for analyses requiring complete parental and family-level information.

### Genotyping and quality control

Genomic DNA from the sister cohort participants was subjected to high-throughput sequencing using the DNBSEQ-T7 platform (MGI Tech Co., Ltd., Shenzhen, China). Raw sequencing reads were aligned to the human reference genome (GRCh37) using BWA-MEM (Li, 2013). Variant calling was performed with GATK HaplotypeCaller (McKenna *et al.*, 2010) following best practices. Standard quality control included filtering SNPs with a call rate <98% and samples with a genotype call rate <95%. Only bi-allelic SNPs were retained for PRS analysis.

Parental genomic DNA from the PGT-M cohort was extracted from peripheral blood. Embryonic DNA was obtained from trophectoderm biopsy cells, followed by whole-genome amplification. All samples were genotyped using the Illumina Asian Screening Array (Illumina, Inc., San Diego, CA, USA). Standard array data quality control was applied: SNPs with a call rate <95% and genotype call rate <90% were excluded. SNPs deviating from Hardy–Weinberg equilibrium (*P* < 1 × 10⁻⁶) in a relevant reference set were also removed. Genotype imputation and haplotype phasing for this cohort were performed using Beagle 5.4 (Browning *et al.*, 2018) with the 1000 Genomes Project Phase 3 as the reference panel, retaining SNPs with an imputation quality (INFO) score >0.8.

### PRS construction and calculation

We applied three established breast cancer PRS models: the 313-variant score (PRS₃₁₃) primarily derived from European ancestry data, and two Asian-optimized scores (PRS₂₆₃ and PRS₁₁₁) (Yang *et al.*, 2022). For each individual across both cohorts, PRS was calculated using PLINK 1.9 (Chang *et al.*, 2015) as the sum of allele dosages weighted by their published effect sizes:


$$\text{PRS}=\sum_{k=0}^{n}\beta_{k}{\text{SNV}}^{k}$$

in which SNV^*k*^ and β_*k*_ represent the allelic dosage and corresponding weight of SNV *k*, and n is the number of SNVs used. To evaluate the applicability of PRS in the local Han Chinese population, we used the previously described sister cohort to assess the model’s performance as a basis for subsequent studies.

To facilitate within-study comparisons, raw PRS values from individuals in the sister cohort and the PGT-M cohort were used to construct a combined reference distribution, and individual PRS values were converted to percentiles (0–100) using this distribution. In the sister cohort, we assessed association with breast cancer status using logistic regression and reported odds ratios (ORs) with 95% CIs. Discrimination was evaluated using the AUROC, calculated along with its 95% CI using the scikit-learn package (Pedregosa *et al.*, 2011) (v 1.6.1) in Python (version 3.10; Python Software Foundation, Wilmington, DE, USA).

### Embryonic PRS inference and family-based analysis

For embryos from PGT-M cycles, embryonic genotypes at all PRS-relevant loci were reconstructed and their PRS values were calculated. This inference leveraged parental high-density genotype data and haplotype information obtained from linkage analysis to deduce allele transmission. Parental PRS percentiles were categorized into three strata based on the risk distribution in the familial validation cohort: low-risk (≤20th percentile), intermediate-risk (21st–79th percentile), and high-risk (≥80th percentile).

### Embryo morphological assessment

Embryo morphology was assessed using the widely adopted Gardner blastocyst grading system (Gardner *et al.*, 2000), which evaluates three key parameters: degree of expansion (1–6), inner cell mass quality (A–C), and trophectoderm quality (A–C). To generate a continuous variable for quantitative analysis and simulation, all transferable blastocysts within a cycle were ranked based on a standardized priority order that primarily considered expansion degree followed by morphological grades (e.g. AA > AB > BA > BB > BC > CB > CC). This ranking yielded a unique morphological priority score for each embryo within each cycle. The detailed, stepwise hierarchy used for converting Gardner grades into this ordinal rank is provided in Supplementary Table S1.

### Simulation framework and assumptions

To explore the potential impact of PES within a PGT-M workflow, we conducted a simulation-based comparison of two embryo selection strategies under a fixed set of modelling assumptions. Within each PGT-M cycle, at each filtering stage, all embryos meeting transfer eligibility criteria were included in the candidate pool for simulation. One embryo per cycle was then selected as the prioritized embryo using predefined rules (highest morphological priority score vs lowest PRS percentile, depending on the strategy). Two selection strategies were simulated: (i) morphology-based embryo prioritization, selecting the embryo with the highest morphological score; and (ii) PRS-based embryo prioritization, selecting the embryo with the lowest PRS percentile. Simulations were conducted under sequential PGT filtering scenarios reflecting clinical workflow: all biopsied blastocysts were first evaluated by PGT-M, and embryos without pathogenic variants were then, where applicable, further assessed by PGT-A to exclude chromosomal aneuploidy. The primary outcome was the difference in PRS percentile of the selected embryo between the two strategies (a hypothesis-generating surrogate endpoint). This outcome was selected to characterize statistical properties of PRS-based ranking; no clinical endpoints or clinical benefit were assessed. Given that small shifts in PRS percentiles may have limited clinical interpretability, PRS percentiles were additionally grouped into three strata: low (≤20th percentile), intermediate (21st–79th percentile), and high (≥80th percentile). Key simulation assumptions are summarized in Supplementary Table S2.

Continuous variables are presented as mean ± standard deviation or median (interquartile range), and categorical variables as frequency (percentage). Group comparisons were performed using *t*-tests, Mann–Whitney *U*-tests, or chi-square tests as appropriate based on data distribution. The relationship between embryo morphological score and PRS was analysed using Spearman’s rank correlation. All statistical analyses were performed using R software (version 4.3.2; R Foundation for Statistical Computing, Vienna, Austria). A two-sided *P*-value < 0.05 was considered statistically significant.

## Results

### Validation of breast cancer PRS models in a local sister-pair cohort

The canonical 313-SNP breast cancer PRS (PRS₃₁₃), originally developed in European populations, has shown robust and reproducible predictive performance across multiple external cohorts, with AUROC values ranging from 0.630 to 0.642. The Asia Breast Cancer Consortium recalibrated the model using East Asian datasets, generating a 263-SNP score (PRS₂₆₃ Asian) with comparable discrimination in regional cohorts (Ho *et al.*, 2022).

To evaluate performance in a within-family context, we assembled a local Han Chinese sister-pair cohort (n = 184). Breast cancer cases (n = 74) were diagnosed at a mean age of 54.1 ± 6.9 years, while unaffected sisters (n = 110) were older than their affected sibling at enrolment (mean age 65.8 ± 4.4 years), which provides a conservative framework for assessing within-family discrimination. Across the three PRS models tested (PRS₃₁₃, PRS₂₆₃ Asian, PRS₁₁₁), performance was consistent with previous population-level estimates (Table 1). As expected in a sibling-pair design, AUROC values were modest. PRS₂₆₃ Asian demonstrated the strongest discrimination (AUROC = 0.60; 95% CI: 0.52–0.68) and clear percentile separation between affected and unaffected sisters (Fig. 1A), providing an empirical estimate of PRS dispersion within shared familial background.

**Figure 1.**
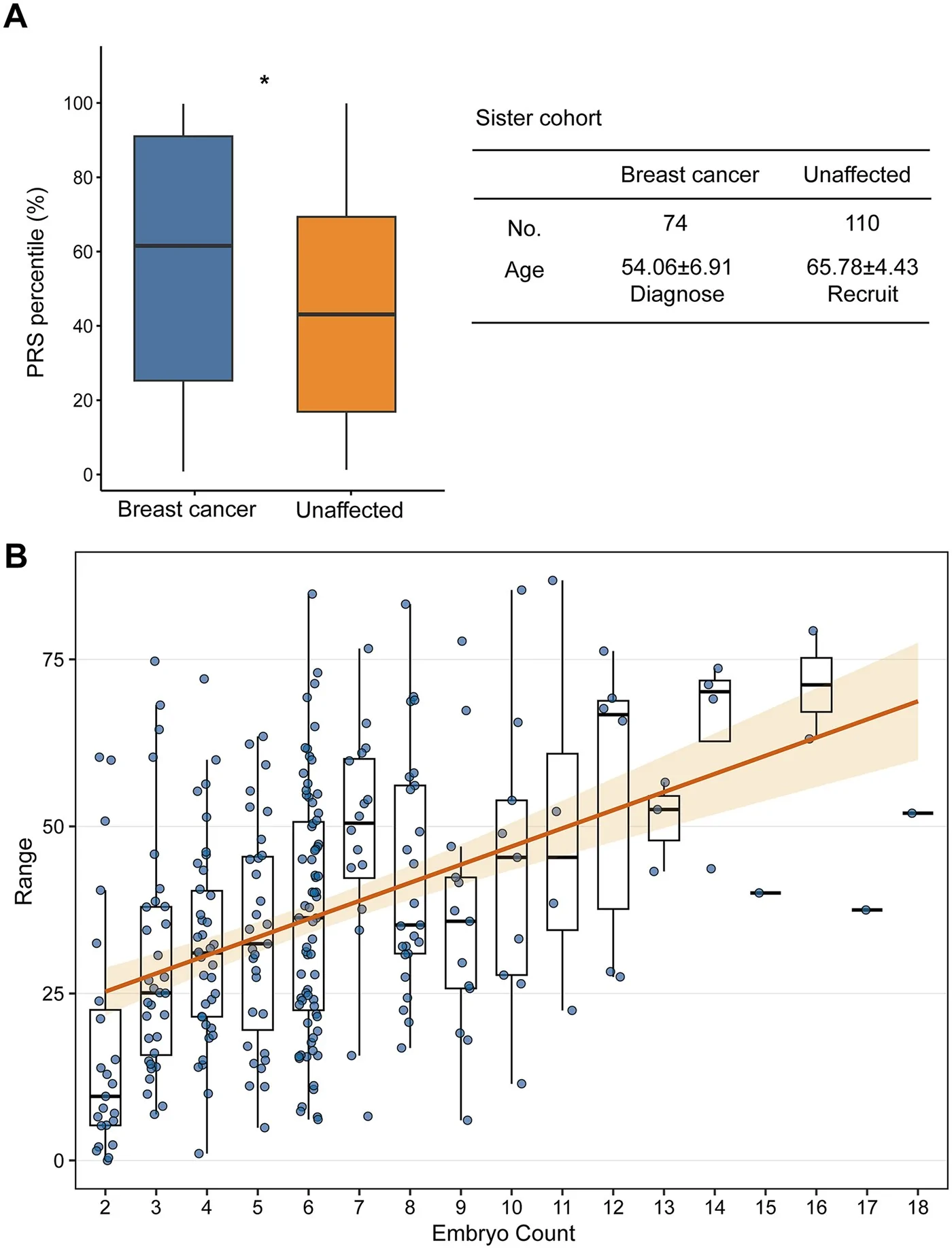
**Within-family breast cancer polygenic risk score distribution and embryo-level PRS variation**. (**A**) Comparison of polygenic risk score (PRS) percentiles between case (n = 74) and control (n = 110) groups in sister pairs. The inset table shows sample size and age (mean ± SD); age refers to age at diagnosis for breast cancer cases and age at recruitment for unaffected sisters. (**B**) Relationship between embryo count and the within-cycle PRS percentile range, calculated as the difference between the maximum and minimum embryo PRS percentiles within each cycle. Each point represents one PGT-M cycle. The orange line represents the fitted linear regression, and the light-yellow shaded area indicates its 95% CI. Boxplots show the median (central line), interquartile range (box), and values within 1.5 times the interquartile range (whiskers). *P* values were derived from Wilcoxon rank-sum tests. *P < 0.05.

**Table 1.** Validation of breast cancer PRS model in local sister cohort.

PRS methods	Odds ratio (95% CI)	AUROC (95% CI)	*P*-value
PRS_313_	1.34 (1.00–1.80)	0.58 (0.51–0.66)	0.05
PRS_263_	1.40 (1.05–1.86)	0.60 (0.52–0.68)	0.02
PRS_111_	1.28 (0.95–1.72)	0.58 (0.50–0.67)	0.1

PRS, polygenic risk score; AUROC, area under the receiver operating characteristic curve.

*P* values were based on Wald tests of the regression coefficients.

Risk stratification was further examined at the extremes of the PRS distribution. Using PRS₂₆₃ Asian, individuals in the top 10% had 4.07-fold higher odds of breast cancer than all others (95% CI: 1.55–6.33; *P *= 0.01). A similar pattern was observed for the top 20% versus the remainder (OR = 3.14; 95% CI: 1.73–9.56; *P *< 0.001) (Table 2 and Supplementary Table S3). These results indicate that, despite the modest AUROC values expected in within-family analysis, the PRS retains the ability to distinguish individuals at the extremes of the polygenic risk distribution.

**Table 2.** Performance of PRS₂₆₃ model in risk stratification.

High PRS_263_ definition	Reference group	Prevalence (%)	Odds ratio (95% CI)	*P*-value
Top 10% of distribution	Remaining 90%	69:30	4.07 (1.73–9.56)	0.01
Top 20% of distribution	Remaining 80%	61:32	3.14 (1.55–6.33)	<0.001

PRS, polygenic risk score.

*P* values were derived from chi-square tests.

### Embryo-level PRS variability within IVF cycles

Given the probabilistic nature of polygenic risk, PES is best conceptualized as ranking rather than excluding embryos. In the full retrospective PGT-M cohort, couples produced a mean of 5.6 blastocysts per cycle, of which 3.1 ± 2.3 were mutation-free and eligible for transfer. Overall, 54.6% of PGT-M cycles yielded ≥3 transferable embryos. This relatively high yield of transferable embryos in PGT-M, compared with PGT-A or PGT-SR, provides a practical context in which PRS-informed ranking can be examined (Table 3 and Supplementary Table S4).

**Table 3.** Comparison of transferable blastocyst yields across PGT types.

PGT type	PGT-A	PGT-SR	PGT-M
Cycle (n)	4382	1005	416
Biopsy/cycle	3.56 ± 2.90	4.50 ± 3.00	4.69 ± 2.73
≥5 blastocysts (%)	28.46	43.18	50.96
Transferable blastocyst/cycle	1.69 ± 1.54	1.68 ± 1.57	3.11 ± 2.26
≥3 Transferable blastocyst cycle (%)	28.4	24.58	54.57

PGT, preimplantation genetic testing; PGT-A, preimplantation genetic testing for aneuploidy; PGT-SR, preimplantation genetic testing for structural rearrangements; PGT-M, preimplantation genetic testing for monogenic disorders.

Using the PRS₂₆₃-Asian model, we analysed the complete-information subset comprising 1722 embryos from 310 PGT-M cycles for family-based and parental PRS analyses. The median within-cycle range of embryonic PRS percentiles was ∼25 points. Furthermore, within-cycle PRS percentile range tended to increase with embryo count (Fig. 1B). These findings demonstrate substantial PRS variability among embryos within the same cycle and suggest that differences in PRS tend to widen as embryo number increases.

### Polygenic background in PGT-M cycles targeting breast cancer susceptibility genes

Accumulating evidence suggests that polygenic background can modify risk variability even in the presence of monogenic susceptibility. We therefore assessed whether polygenic background could provide additional context in cycles targeting breast cancer susceptibility genes.

Comparison of PRS₂₆₃ distributions between embryos from PGT-M cycles targeting breast cancer susceptibility genes (n = 25) and embryos from other cycles revealed the absolute difference-in-median-PRS values between groups were minimal (Fig. 2A).

**Figure 2.**
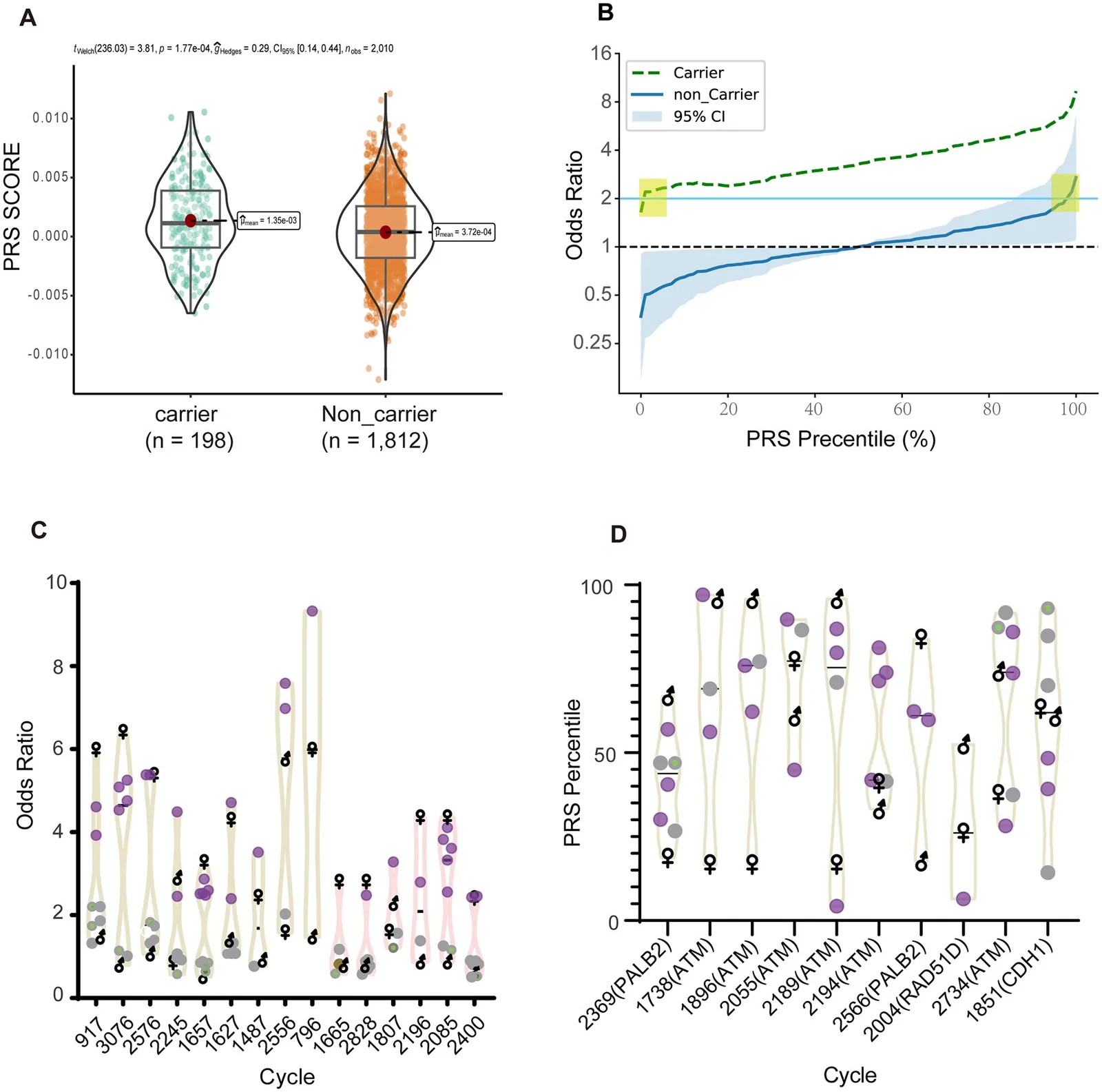
**Polygenic embryo screening (PES) in preimplantation genetic testing for monogenic disorders (PGT-M) cycles involving breast cancer susceptibility genes**. (**A**) Distribution of polygenic risk score (PRS) values among embryos stratified by carrier status of breast cancer-related pathogenic variants in the PGT-M cohort. Comparison of PRS₂₆₃ distributions between carrier (n = 198) and non-carrier (n = 1812) embryos in the full PGT-M embryo dataset. *P* values were derived from Welch’s *t*-tests; effect sizes are reported as Hedges’ g (${\hat{g}}_{\mathrm{Hedges}}$). The estimated overall pooled mean (μ̂_mean_). is indicated above the plot Boxplots display medians and interquartile ranges (IQR); whiskers extend to the most extreme values within 1.5 × IQR from the box edges, with points beyond shown as outliers. Boxplots display medians and interquartile ranges, with each point representing an individual embryo. (**B**) Estimated odds ratios (OR) across PRS percentiles for BRCA1/2 pathogenic variant carriers (green dashed line) and non-carriers (blue solid line). Shaded areas indicate 95% CIs. (**C**) PES outcomes in BRCA1/2 PGT-M cycles. Embryo-specific breast cancer ORs were derived using the model shown in panel B. Within each cycle, embryos are ranked by PRS percentile. The *y*-axis represents estimated ORs, and the *x*-axis indicates individual PGT-M cycles. Symbols (♂ ♀) denote parental samples; purple dots represent carrier embryos, grey dots represent non-carrier embryos, and green highlights indicate the morphologically highest-quality embryo within each cycle. Pale yellow and pink violin outlines denote BRCA1 and BRCA2 cycles, respectively. (**D**) PES results in PGT-M cycles involving other breast cancer susceptibility genes. Due to limited research data, OR estimation was not performed; instead, embryo-specific PRS percentile rankings are shown. The *y*-axis represents PRS percentile ranks, and the *x*-axis denotes individual PGT-M cycles and corresponding genes. Symbols (♂ ♀), purple dots, grey dots and green highlights indicate as in panel C. Pale violin outlines show the within-cycle distribution of embryo PRS percentiles.

*BRCA1/2* mutations confer a lifetime breast cancer risk of ∼69–72% (Kuchenbaecker *et al.*, 2017). Large-scale modelling studies combining monogenic and polygenic risk (Lakeman *et al.*, 2021) suggest that, while *BRCA1/2* mutations remain the primary determinant of risk, polygenic background may introduce modest variability. This effect is most apparent at the extremes of the PRS distribution. For instance, a *BRCA1/2* carrier in the lowest 5th PRS percentile may approach the predicted risk of a non-carrier in the highest 95th percentile (Fig. 2B).

In our retrospective analysis of *BRCA1/2* PGT-M cycles (n = 15), embryos carrying the familial pathogenic variant consistently exhibited higher predicted ORs than non-carriers within the same cycle (Fig. 2C). Nonetheless, variability among embryos was observed. In Cycle 1627, two *BRCA1* carrier embryos differed by 2.1-fold in predicted OR, whereas non-carrier embryos showed limited variability. Across families, the mean predicted OR of *BRCA1/2* carrier embryos differed by up to 2.8-fold between Cycles 2556 and 1627. This analysis was descriptive and intended to illustrate modelled heterogeneity arising from the combined contribution of monogenic and polygenic risk. It should not be interpreted as supporting embryo selection among BRCA1/2 carrier embryos, for which pathogenic variant status remains the primary determinant of embryo classification and transfer eligibility in standard PGT-M practice.

For moderate-penetrance genes such as *ATM* and *CHEK2*, estimated lifetime risks are lower (∼25–30%). As robust composite monogenic–polygenic risk models remain limited, we examined PRS percentile distributions in embryos from 10 PGT-M families targeting these genes (Fig. 2D). Notably, some carrier embryos fell within the lowest PRS percentiles (e.g. Cycle 2189), whereas some non-carrier embryos ranked within the highest percentiles (e.g. Cycle 2734). Given that *ATM* mutations confer a more modest increase in risk (OR of ∼2–3), PRS-defined risk distributions of carrier and non-carrier embryos may show greater overlap.

Collectively, these results suggest that monogenic status remains the primary determinant of embryo-level classification in PGT-M cycles, while PRS contributes additional variability among embryos. For moderate-penetrance genes, both carrier and non-carrier embryos were observed across extreme PRS percentiles, corresponding to overlapping ranges of estimated risk.

### Parental PRS stratification as a key consideration for embryo ranking

Incorporating PES into PGT-M cycles not primarily driven by polygenic disease risk may introduce additional complexity in decision-making (Siermann *et al.*, 2022a), as embryo-level PRS differences are shaped by parental genetic background. Correlation analyses confirmed a significant association between embryo PRS and mean parental PRS, consistent with the additive (polygenic) inheritance (Fig. 3A).

**Figure 3.**
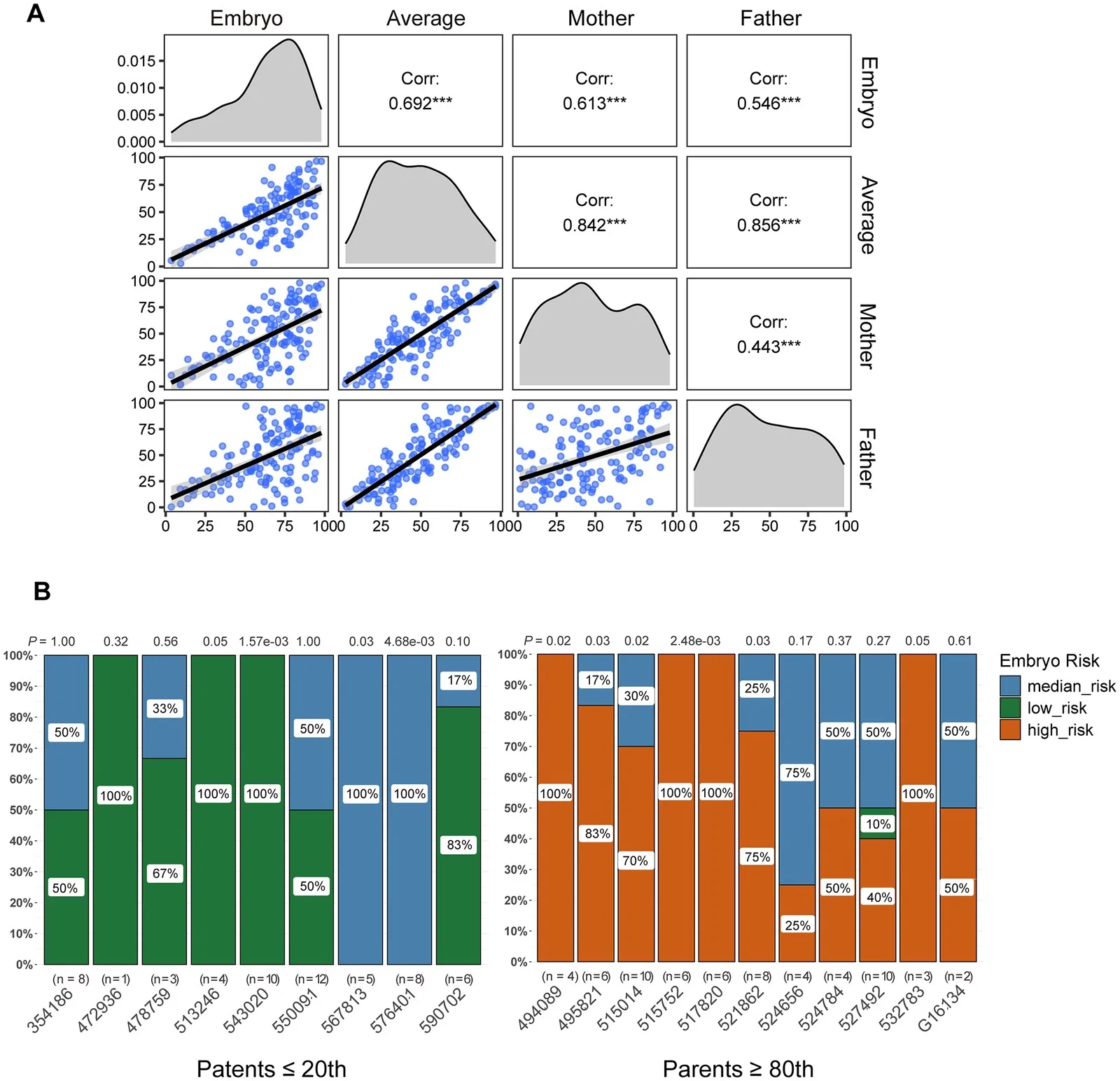
**Relationship of polygenic risk score (PRS) percentiles between embryos and their parents**. (**A**) Relationships of PRS percentiles between embryos and their parents. Diagonal panels display the marginal density distributions of PRS percentiles. Lower, left triangle panels show scatter plots with locally estimated scatterplot smoothing (LOESS) curves and 95% CI (grey bands). Upper, right triangle panels report Pearson correlation coefficients, with *P* values derived from correlation significance tests. ***P <0.001. (**B**) Distribution of embryos across PRS risk categories stratified by parental PRS status. Embryos are grouped according to whether both parents fall into the low-PRS (≤20th percentile) or high-PRS (≥80th percentile) strata. Values within columns indicate the percentage of embryos in each PRS stratum; n indicates the number of embryos in the family. *P* values above the columns indicate the significance of differences in risk stratum distribution across embryo groups, and were calculated using Fisher’s exact test.

Using percentile-based PRS strata derived from the sister-pair validation cohort (≤20th, 21st–79th, ≥80th), we examined how parental PRS background shapes embryo-level PRS distributions (Table 4; Supplementary Fig. S1). When at least one parent was in the low-PRS stratum (≤20th percentile), the probability of generating an embryo in the high-PRS stratum (≥80th) was low (7.37%; 95% CI: 5.72–9.45%). In contrast, when both parents were ≥80th percentile, most embryos also fell within the high-PRS stratum (71.4%; 95% CI: 59.3–81.1%) (Fig. 3B).

**Table 4.** Expected embryo PRS distribution under different parental PRS combinations.

Parents status	Embryos status		
RISK	Reduced (95% CI) (%)	Median (95% CI) (%)	Elevated (95% CI) (%)
One ≤20%	35.79 (32.46–39.26)	56.84 (53.30–60.32)	7.37 (5.72–9.45)
Both ≤20%	65.65 (57.18–73.23)	34.35 (26.77–42.83)	–
One ≤20%, One 20–80%	31.96 (28.21–35.94)	59.79 (55.66–63.78)	8.26 (6.25–10.84)
One ≤20%, One ≥80%	11.27 (5.82–20.69)	74.65 (63.45–83.32)	14.09 (7.83–24.02)
Both 20–80%	7.75 (5.90–10.13)	69.95 (66.23–73.43)	22.29 (19.19–25.74)
One 20–80%, One ≥80%	1.15 (0.39–3.32)	56.71 (50.64–62.58)	42.15 (36.31–48.21)
Both ≥80%	1.59 (0.28–8.46)	26.98 (17.58–39.03)	71.43 (59.30–81.10)

PRS, polygenic risk score.

These patterns indicate that parental PRS profiles constrain the distribution of embryo-level PRS. Under high parental PRS backgrounds, embryo PRS values were generally higher, reducing the likelihood of identifying embryos at the lower extreme of the distribution, while still allowing a degree of within-cycle variation.

### Impact of PRS-prioritized selection in PGT-M embryos

In routine clinical practice, embryo morphological grading remains an important criterion for transfer prioritization. In our cohort, Spearman correlation analysis showed no significant association between embryo morphology score and breast cancer PRS (correlation coefficient = 0.18; *P *= 0.42) (Supplementary Fig. S2), indicating that morphological quality and polygenic risk represent largely independent parameters.

To evaluate the potential impact of PES on PRS-based embryo ranking, we simulated PRS-based embryo prioritization and compared it with morphology-based embryo prioritization. Embryo PRS percentiles were examined at three sequential stages—post-blastocyst biopsy, after PGT-M, and after combined PGT-M with  PGT-A—to assess how sequential testing shapes polygenic risk distributions among transferable embryos. Across all stages, PRS-prioritized selection consistently yielded lower mean PRS percentiles than morphology-based selection. However, the magnitude of the difference decreased as embryos were sequentially filtered for monogenic variants and chromosomal abnormalities, reaching approximately a 10-percentile-point difference at the final transferable-embryo stage (Fig. 4A–C). This magnitude reflects a modelled ranking difference only and should not be interpreted as a demonstrated reduction in lifetime breast cancer risk or other clinical benefit. This attenuation coincided with progressive reductions in embryo availability after genetic filtering, resulting in fewer embryos available for within-cycle prioritization.

**Figure 4.**
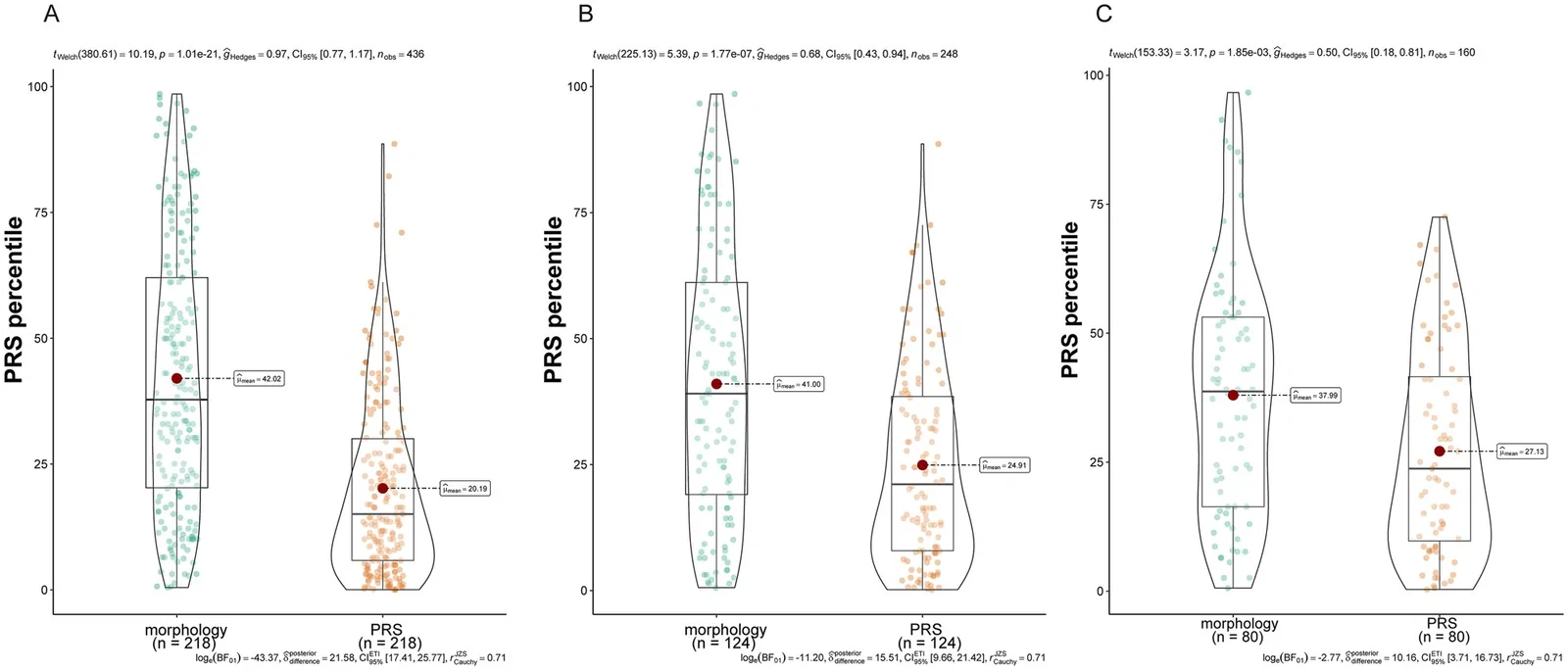
**Comparison of embryo selection strategies based on morphology-prioritized and polygenic risk score (PRS)-prioritized approaches**. (**A**) All embryos generated per cycle, representing the maximal set of available embryos. (**B**) Embryos eligible for transfer after preimplantation genetic testing for monogenic disorders (PGT-M), excluding those carrying monogenic pathogenic variants. (**C**) Embryos eligible for transfer after combining PGT-M and preimplantation genetic testing for aneuploidy (PGT-A), excluding embryos with monogenic pathogenic variants and those with aneuploidy. Across all panels, the *y*-axis represents PRS percentiles; green denotes embryos selected under morphology-prioritized strategy, and orange denotes those selected under PRS-prioritized strategy. Progressive filtering across panels reduces the pool of available embryos and attenuates the magnitude of PRS percentile shifts, highlighting the dependence of relative prioritization effects on the underlying selection constraints. Boxplots show the median (central line), interquartile range (IQR), and whiskers extending to 1.5 × IQR; points beyond are shown as outliers. Statistical values displayed above the plots include: Welch’s *t*-test statistic (*t*_welch_), *P*-value (*p*), Hedges’ g effect size (${\hat{g}}_{\mathrm{Hedges}}$) with its 95% CI, and number of observations (n_obs_), and estimated overall pooled mean (μ̂_mean_). Bayesian statistics include the natural logarithm of the Bayes factor (Log_e_(BF_01_)), the posterior mean of the difference (${\hat{\delta }}_{\mathrm{difference}}^{\mathrm{postterior}}$), and the 95% equal-tailed interval [${\mathrm{CI}}_{95\%}^{\mathrm{ETI}}$], with the JZS Cauchy prior scale ($r_{\mathrm{Cauchy}}^{\mathrm{JZS}}$).

Finally, we examined whether PRS-prioritized embryo selection alters categorical risk strata across parental PRS backgrounds, as shifts in PRS percentiles alone may be difficult to interpret in isolation given the imprecision of current models. In families where at least one parent had elevated polygenic risk (≥80th percentile), or both parents were in the intermediate-risk range, PRS-prioritized ranking occasionally shifted the leading transferable embryo from a high- to an intermediate-PRS stratum. When neither parent was classified as high risk, PRS-prioritized selection occasionally shifted the leading transferable embryo into the low-PRS stratum (Supplementary Figs S3, S4, and S5). However, such shifts occurred in only a subset of families, and in most cases PRS strata remained unchanged.

## Discussion

In this study, we conducted a context-specific evaluation of PES retrospectively by applying it to embryos from PGT-M cycles. Because PES involves ranking embryos that share the same parental genome, its feasibility depends on whether PRS varies sufficiently among embryos within families; the sister-pair analysis was therefore performed as an empirical approximation of this within-family PRS variation. Our findings are pragmatic: PES is associated with modest shifts in PRS percentile distributions among transferable embryos, and the magnitude of these shifts depends strongly on clinical context. The largest differences are observed in best-prognosis cycles—those yielding multiple transferable embryos—and when one or both parents fall within elevated PRS strata. Conversely, when embryo numbers are limited or the risk landscape is dominated by high-penetrance monogenic variants, the scope for PRS-based prioritization is limited.

PRS represents probabilistic susceptibility rather than a deterministic outcome and should not be interpreted as categorizing embryos into ‘healthy’ versus ‘affected’. The realistic objective of PES is to avoid selecting the comparatively highest-risk embryos rather than to identify embryos with near-zero genetic risk. In our cohort, cycles yielding three or more transferable embryos exhibited an average within-cycle spread of approximately 25 percentile points, and the within-cycle range tended to increase with the number of embryos per cycle. This within-family variation provides the statistical basis for embryo ranking and helps explain why PES is most relevant in PGT cycles with a favourable prognosis. When only one or two embryos are available, the scope for meaningful PRS-based prioritization is inherently limited.

The balance between monogenic and polygenic components further delineates the setting in which PES may add information. For high-penetrance variation, the monogenic effect dominates projected lifetime risk and produces risk distributions that are largely non-overlapping with those of non-carriers across most PRS percentiles (Kuchenbaecker *et al.*, 2017), as observed in our *BRCA1/2* cycles. In contrast, for moderate-penetrance genes such as *ATM* or *CHEK2*, polygenic background may contribute meaningfully to overall risk variation, particularly at the extremes of the PRS distribution (Breast Cancer Association Consortium *et al.*, 2021). Modelling studies suggest that, for moderate-penetrance variants, PRS may occasionally contribute to overlapping estimated risk ranges between carriers and non-carriers (Lencz *et al.*, 2026). Translating these observations into clinical decision-making would require integrated models combining monogenic and polygenic risk on a common calibrated scale and validation across ancestrally matched populations (Manolio *et al.*, 2019). Current limitations—including uncertain penetrance for moderate-penetrance variants, small or population-specific cohorts, variable PRS transferability, and lack of prospective validation—mean such approaches remain investigational.

Although each locus contributing to a polygenic score follows Mendelian inheritance, the aggregation of hundreds of variants complicates intuitive estimation of transmission and embryo-level PRS distributions. Parental PRS stratification illustrates a probabilistic inheritance pattern analogous to recurrence-risk reasoning in monogenic counselling. Our analyses show that when both parents fall within high PRS strata, the probability of generating an embryo in the high-PRS stratum increases. Importantly, disclosure of polygenic risk may itself introduce psychological burden, a challenge widely recognized in the clinical implementation of PRS (Lewis and Vassos, 2020). Evidence from PRS-informed risk assessment indicates that structured pre-test counselling, explicit informed consent, and linkage to actionable management strategies—such as risk-stratified breast cancer screening—are essential safeguards (Martínez-Minguet *et al.*, 2026). Within this framework, parental PRS information may help contextualize embryo-level variation in exploratory PES analyses, but careful communication remains critical to avoid increasing anxiety or unrealistic expectations.

In our transfer simulations, after excluding embryos affected by monogenic or chromosomal abnormalities, PES produced an approximate 10-percentile shift in the PRS percentiles of the selected embryo. Such shifts typically correspond to modest modelled differences in relative risk across the central portion of the PRS distribution, where risk gradients are relatively shallow (Fig. 2B). In practical terms, these differences are more interpretable when they result in movement between broader PRS strata rather than small numerical changes in PRS percentile. Consistent with this interpretation, PRS-based prioritization occasionally shifts the leading transferable embryo from a higher-PRS to a lower-PRS stratum—for example, from a higher-PRS to an intermediate-PRS stratum in families with elevated parental PRS (Supplementary Figs S3, S4, and S5). These observations provide a framework for interpreting percentile shifts, although the clinical significance remains uncertain. Importantly, the observed ∼10-percentile shift is a surrogate outcome from retrospective modelling. No clinical benefit or reduction in disease incidence was demonstrated; any interpretation in terms of ‘risk reduction’ should be limited to statistical projections within the model.

This study is retrospective, focuses on a single disease model, and is restricted to best-prognosis PGT-M cycles that are not representative of the broader IVF population. The moderate AUROC values observed in sibling-based validation reflect the constrained genetic variance inherent to related samples and should not be interpreted as the primary metric of embryo-level utility. In the context of PGT workflows, the relevant question is not population-level discrimination but the ability to support within-cycle prioritization among available embryos. Central to interpretation, this study did not evaluate clinical endpoints; all modelled risk estimates are statistical projections and have not been validated as predictors of offspring disease outcomes. Whether such prioritization translates into meaningful differences in lifetime disease incidence, patient experience, or cost-effectiveness remains unknown and will require prospective longitudinal studies with clinical follow-up.

Our findings characterize the statistical properties of PRS-based embryo ranking in a specific best-prognosis PGT-M context. Where applicable, PES may offer limited, context-dependent information only as a possible adjunct within established PGT workflows, that is, to rank embryos after monogenic and chromosomal exclusions, not as a stand-alone screening test. These findings do not establish clinical utility, demonstrate reduction in disease incidence, or provide a basis for implementation outside a prospective research framework with full ethical oversight and informed consent. Accordingly, premature translation into routine clinical practice risks causing harm disproportionate to any plausible benefit.

Given that PES is already offered commercially in some settings, premature translation into routine practice remains a particular concern. In the absence of validated embryo-level utility, wider adoption may increase parental anxiety, widen inequitable access, and create pressure to rank embryos based on polygenic predictions of limited validity. Accordingly, these findings should not be interpreted as supporting routine or standalone clinical use of PES. Rather, they provide exploratory evidence to inform ongoing ethical, methodological, and clinical discussions and to caution against implementation ahead of adequate validation.

## Supplementary Material

hoag078_Supplementary_Data

## Data Availability

The data underlying this article will be shared on reasonable request to the corresponding author.
