# Supplementary material for: A context-specific evaluation of polygenic embryo screening in best-prognosis preimplantation genetic testing cycles
Source: Hum Reprod Open. 2026 Sep 8;2026(4):hoag078. doi: 10.1093/hropen/hoag078 (PMC13630452; doi:10.1093/hropen/hoag078)
Supplement: Supplementary file 1 [file hoag078_supplementary_data.docx]

**Supplementary Data**

**A context-specific evaluation of polygenic embryo screening in best-prognosis preimplantation genetic testing cycles**

Qi Wang, SiCong Zeng, ZiXu Chen, Liang Hu, Xiao Hu, PingYuan Xie, Juan Du, XiLin Xu, GuangXiu Lu, YueQiu Tan, Ge Lin

**Table of Contents**

**Supplementary Figure S1.** Distribution of embryo PRS risk categories under different parental PRS stratifications **Supplementary Figure S2.** Association between embryo morphological grade and PRS percentile **Supplementary Figure S3.** Embryo PRS percentiles under morphology-prioritized vs. PRS-prioritized approaches (All embryos) **Supplementary Figure S4.** Embryo PRS percentiles under morphology-prioritized vs. PRS-prioritized approaches (PGT-M—eligible embryos) **Supplementary Figure S5.** Embryo PRS percentiles under morphology-prioritized vs. PRS-prioritized approaches (PGT-M/PGT-A—eligible embryos)

**Supplementary Table S1.** Embryo morphological grading hierarchy and corresponding priority ranking used in simulation analyses

**Supplementary Table S2.** Summary of simulation framework and key assumptions.
**Supplementary Table S3.** Association between PRS percentiles and estimated odds ratios for clinical outcomes in the sister cohort **Supplementary Table S4.** Statistical summary of embryo counts and corresponding family frequencies

**Supplementary Figure S1 Distribution of embryo PRS risk categories under different parental PRS stratifications.**

Parental PRS levels were classified into high (≥80th percentile), moderate (20th–80th percentile), and low (≤20th percentile) categories. The figure illustrates how different combinations of parental PRS risk categories influence the distribution of PRS risk categories among embryos.

**Supplementary** **Figure S2 Association between embryo morphological grade and PRS percentile.**

No significant association was observed. Each dot represents an embryo from the PGT-M cohort.

PRS, polygenic risk score; PGT-M, preimplantation genetic testing for monogenic disorders.

**Supplementary Figure S3 Embryo PRS percentiles under morphology-prioritized vs. PRS-prioritized approaches (All embryos).**

Across the full embryo set, including those with monogenic mutations, breast cancer PRS percentiles are compared between morphology-prioritized (red) and PRS-prioritized (blue) strategies under different parental PRS backgrounds. Boxplots show the median (central line), interquartile range (IQR), and whiskers extending to the most extreme values within 1.5×IQR from the box edges; points beyond are shown as outliers. P values were derived from paired t-tests. ^*^*P* < 0.05 ^***^*P*< 0.001 ^****^*P*< 0.0001.

PRS, polygenic risk score.

**Supplementary** **Figure S4 Embryo PRS percentiles under morphology-prioritized vs. PRS-prioritized approaches (PGT-M—eligible embryos).**

After excluding embryos with monogenic mutations, PRS percentiles are compared between the two strategies across different parental PRS backgrounds. Boxplots show the median (central line), interquartile range (IQR), and whiskers extending to the most extreme values within 1.5×IQR from the box edges; points beyond are shown as outliers. P values were derived from paired t-tests.^***^*P*< 0.001 ^****^*P*< 0.0001.

PRS, polygenic risk score; PGT-M, preimplantation genetic testing for monogenic disorders.

**Supplementary** **Figure S5 Embryo PRS percentiles under morphology-prioritized vs. PRS-prioritized approaches (PGT-M/PGT-A—eligible embryos)**

After excluding embryos with monogenic pathogenic variants and aneuploid embryos, PRS percentiles are compared between the two strategies across different parental PRS backgrounds. Boxplots show the median (central line), interquartile range (IQR), and whiskers extending to the most extreme values within 1.5×IQR from the box edges; points beyond are shown as outliers. P values were derived from paired t-tests. ^**^*P*< 0.01 ^***^*P*< 0.001.

PRS, polygenic risk score; PGT-M, preimplantation genetic testing for monogenic disorders; PGT-A, preimplantation genetic testing for aneuploidy.

Supplementary Table S1. Embryo morphological grading hierarchy and corresponding priority ranking used in simulation analyses.

| **Priority Rank** | **Morphological Grade** |
| --- | --- |
| 1 | 5-6AA |
| 2 | 5-6BA |
| 3 | 5-6AB |
| 4 | 5-6BB |
| 5 | 5-5AA |
| 6 | 5-5BA |
| 7 | 5-5AB |
| 8 | 5-5BB |
| 9 | 5-4AA |
| 10 | 5-4BA |
| 11 | 5-4AB |
| 12 | 5-4BB |
| 13 | 6-6AA |
| 14 | 6-6BA |
| 15 | 6-6AB |
| 16 | 6-6BB |
| 17 | 6-5AA |
| 18 | 6-5BA |
| 19 | 6-5AB |
| 20 | 6-5BB |
| 21 | 6-4AA |
| 22 | 6-4BA |
| 23 | 6-4AB |
| 24 | 6-4BB |
| 25 | 5-6AC |
| 26 | 5-6BC |
| 27 | 5-5AC |
| 28 | 5-5BC |
| 29 | 5-4AC |
| 30 | 5-4BC |
| 31 | 5-6CA |
| 32 | 5-6CB |
| 33 | 5-5CA |
| 34 | 5-5CB |
| 35 | 5-4CA |
| 36 | 5-4CB |
| 37 | 6-6AC |
| 38 | 6-6BC |
| 39 | 6-5AC |
| 40 | 6-5BC |
| 41 | 6-4AC |
| 42 | 6-4BC |
| 43 | 6-6CA |
| 44 | 6-6CB |
| 45 | 6-5CA |
| 46 | 6-5CB |
| 47 | 6-4CA |
| 48 | 6-4CB |
| 49 | 7-6AA |
| 50 | 7-6BA |
| 51 | 7-6AB |
| 52 | 7-6BB |
| 53 | 7-5AA |
| 54 | 7-5BA |
| 55 | 7-5AB |
| 56 | 7-5BB |
| 57 | 7-4AA |
| 58 | 7-4BA |
| 59 | 7-4AB |
| 60 | 7-4BB |
| 61 | 7-6AC |
| 62 | 7-6BC |
| 63 | 7-5AC |
| 64 | 7-5BC |
| 65 | 7-4AC |
| 66 | 7-4BC |
| 67 | 7-6CA |
| 68 | 7-6CB |
| 69 | 7-5CA |
| 70 | 7-5CB |
| 71 | 7-4CA |
| 72 | 7-4CB |

Format: [Day of Development]- [Gardner Score]. Embryos were ranked from highest (Rank 1) to lowest priority based primarily on day of development (Day 5 > Day 6 > Day 7), followed by standard Gardner blastocyst grading (AA, AB, BA, BB, then AC/BC, CA/CB).

Supplementary Table S2. Summary of simulation framework and key assumptions.

| **Feature** | **Description** |
| --- | --- |
| **Study population** | Simulations were restricted to PGT-M cycles yielding ≥3 embryos, reflecting best-prognosis cases. |
| **Sequential filtering** | Embryos were filtered sequentially based on monogenic variant status (PGT-M) and, where applicable, chromosomal aneuploidy (PGT-A), consistent with the three stages analyzed in the results. |
| **Embryo availability** | The number of embryos available for prioritization was empirically derived from the observed cohort and progressively decreased across filtering steps. |
| **Ranking-only strategy** | PRS was used exclusively for relative prioritization among transferable embryos; embryos were not excluded on the basis of PRS alone. |
| **Outcome metric** | Effect size was defined as the difference in PRS percentile of the selected embryo. In addition, categorical risk strata (low/intermediate/high) were assessed to evaluate practical shifts in projected risk. |
| **parental PRS stratification** | Simulations were stratified by predefined parental PRS background (e.g., both are ≥80th percentile) to explore context-dependent effects on embryo-level risk. |

PRS, polygenic risk score; PGT-M, preimplantation genetic testing for monogenic disorders; PGT-A, preimplantation genetic testing for aneuploidy.

Supplementary Table S3 Association between PRS percentiles and estimated odds ratios for clinical outcomes in the sister cohort.

| **Group** | **OR** | **CI_lower** | **CI_upper** |
| --- | --- | --- | --- |
| **0-20^th^** | 0.6460 | 0.4358 | 0.9574 |
| **20-40^th^** | 0.8518 | 0.7372 | 0.9842 |
| **40-60^th^** | 1.0000 | 1.0000 | 1.0000 |
| **60-80^th^** | 1.1745 | 1.0161 | 1.3576 |
| **80-100^th^** | 1.5500 | 1.0446 | 2.3000 |

PRS values were stratified into five quantile-based groups: 0–20^th^, 20–40^th^, 40–60^th^, 60–80^th^, and 80–100^th^ percentiles. The middle group (40–60th percentile) was used as the reference for Odds ratios (OR) calculation. Odds ratios and 95% confidence intervals (CI) were estimated for each group using the logistic regression coefficient and its variance, based on the deviation of the group median PRS from the overall median.

Supplementary Table S4: Statistical Summary of Embryo Counts and Corresponding Family Frequencies.

| **Embryo Count** | **Family Count** |
| --- | --- |
| 2 | 24 |
| 3 | 32 |
| 4 | 38 |
| 5 | 32 |
| 6 | 71 |
| 7 | 16 |
| 8 | 25 |
| 9 | 13 |
| 10 | 9 |
| 11 | 4 |
| 12 | 6 |
| 13 | 3 |
| 14 | 4 |
| 15 | 1 |
| 16 | 2 |
| 17 | 1 |
| 18 | 1 |
